# Health service utilization among African American women living with systemic lupus erythematosus: perceived impacts of a self-management intervention

**DOI:** 10.1186/s13075-019-1942-7

**Published:** 2019-06-25

**Authors:** Abena A. Twumasi, Anna Shao, Charmayne Dunlop-Thomas, Cristina Drenkard, Hannah L. F. Cooper

**Affiliations:** 1 Department of Behavioral Sciences and Health Education, Emory University Rollins School of Public Health, Atlanta, GA USA; 2 Department of Medicine, Emory University School of Medicine, Atlanta, GA USA

**Keywords:** Systemic lupus erythematosus, Health service utilization, Qualitative methods

## Abstract

**Background:**

Healthcare access, utilization, and quality play critical roles in shaping mortality and morbidity among patients diagnosed with systemic lupus erythematosus (SLE), and yet healthcare access, utilization, and quality can be suboptimal for many people living with SLE. The aim of this qualitative study was to explore the perceived impact of a peer-led, group-based educational intervention (the Chronic Disease Self-Management Program [CDSMP]) on healthcare engagement behaviors among African American women with SLE.

**Methods:**

Participants were recruited from the WELL (Women Empowered to Live with Lupus) study, a behavioral trial of the effectiveness of the CDSMP on African American women diagnosed with SLE. We conducted two waves of qualitative, one-on-one, semi-structured interviews with 24 purposively sampled WELL participants; one interview was conducted before CDSMP participation and one after. Wave 1 interviews explored health service use behaviors at baseline; Wave 2 interviews focused on changes in these behaviors post-intervention and women’s perceptions of whether and how the CDSMP shaped these changes. Transcripts were analyzed using thematic analysis methods.

**Results:**

Study participants perceived the CDSMP to be a valuable resource for supporting two distinct health service use behaviors: communicating with doctors (*N* = 16 [88.9%]) and managing medication side effects (*N* = 17 [41.2%]). Women perceived that the CDSMP had the most potent and widespread effects on patients’ communication with doctors. Strategies that women believed generated improvements in patient-doctor communication included enhancing preparation for appointments and boosting patient participation during doctor’s visits. Women’s reported post-CDSMP improvements in health service use behaviors varied by disease severity and depression. Insurance coverage, while not probed directly during baseline interviews, emerged organically as a key factor affecting health service use behaviors; the CDSMP did not seem to improve participants’ ability to circumvent insurance-related barriers to accessing care.

**Conclusions:**

Our findings suggest that the CDSMP may help enhance healthcare service utilization among African American women with SLE by improving doctor/patient communication and medication side effect management. If future research confirms this conclusion, African American women living with SLE should be encouraged to participate in CDSMP workshops to enhance health service use behaviors.

**Trial registration:**

NCT02988661. Registered 12/07/2016

**Electronic supplementary material:**

The online version of this article (10.1186/s13075-019-1942-7) contains supplementary material, which is available to authorized users.

## Introduction

Systemic lupus erythematosus (SLE) is a chronic autoimmune condition characterized by a broad spectrum of clinical manifestations, including, pain, skin rashes, arthritis, and damage to vital internal organ systems [1]. Although therapeutic advances in recent years have led to improved health outcomes among SLE patients [2], racial/ethnic disparities persist [3]. Compared to White adults, African American adults have a threefold higher incidence and prevalence of SLE [4–7], experience more severe disease activity, and have a worse prognosis [8, 9]. African American *women* are especially vulnerable to SLE morbidity and mortality: SLE prevalence is 10- to 15-fold higher in women compared to that in men [4–7].

Healthcare access, healthcare use, and quality of healthcare play a critical role in disease outcomes and health disparities among patients with SLE [3, 10–13]. For example, patients with SLE who reported poor patient-provider communication and poor care coordination showed significantly greater accrual of disease damage over time [14]; conversely, SLE patients who actively engaged clinicians during consultations showed less organ damage over a nearly 5-year median follow-up period [15]. Unfortunately, significant racial/ethnic inequities exist in healthcare use among SLE patients: an analysis of hospital and emergency room (ER) admissions over a 13-year period for incident SLE patients from the Georgia Lupus Registry (GLR) revealed that African American adults had a significantly higher rate of hospital and ER admissions compared to White adults [10].

Educational self-management programs are integral to the chronic disease standard of care [16–19] and may help patients better navigate healthcare services. These programs aim to empower individuals in the utilization of social support resources to optimize health and reduce the impact of disease on daily functioning [20]. Self-management education is a process helping patients to bridge the gap between disease knowledge and health-related behaviors [21]. Evidence-based self-management education programs have been designed to increase patients’ skills and confidence in managing their symptoms and medications, as well as to teach patients how to effectively communicate with healthcare providers and navigate the healthcare system [22–25]. While the self-management needs, goals, and strategies of each patient may vary, Lorig has identified five core skills that enhance self-management: (a) problem solving, (b) decision making, (c) resource utilization, (d) formation of patient-clinician partnerships, and (e) taking action [25, 26].

A growing body of evidence suggests that in addition to improving health-related behaviors and disease outcomes, self-management programs can also impact healthcare utilization in people with chronic conditions [27]. For instance, findings from a longitudinal nationwide study reported 5% reduction in ER visits at both 6 and 12 months, and 3% reduction in hospitalizations at 6 months [28, 29]. While these and other studies suggest that generic self-management interventions may contribute to improve healthcare service use, their findings may not generalize to people living with lupus because these studies often sampled older and White participants. In contrast, lupus patients tend to be younger and African American, and may thus face unique psychosocial barriers to healthcare service use. For instance, socioeconomically disadvantaged patients with lupus and other rheumatic diseases are likely to experience treatment non-adherence, financial problems, fear of side effects, difficulty navigating the health system, and perceived treatment inefficacy [30].

Currently, there are no widely available, evidence-based, SLE-specific self-management resources. The SLE Self-Help Course (SLESH) was the only SLE-specific self-management intervention in the USA in the 1990s [31]. Despite promising results and valiant effort by the Arthritis Foundation to disseminate SLESH across the USA, the program has not been available since the late 1990s (Personal communication with J Galloway, on July 3, 2012). One challenge of SLESH in particular is the relatively significant effort needed to implement and disseminate the program in light of the relatively low prevalence of SLE. Thus, effective self-management in SLE comes from what patients acquire on their own or from their providers.

The present longitudinal, qualitative study is designed to explore women’s perceptions of the ways that one self-management program, the Chronic Disease Self-Management Program (CDSMP), affects healthcare service engagement among African American women living with SLE. The CDSMP, a globally recognized, evidence-based self-management intervention, is a group-based workshop designed to foster self-efficacy and encourage patients to actively partner with their healthcare providers to optimize their care [24]. Because the CDSMP is a cost-effective, nationally available intervention, it could potentially facilitate reduction in use of healthcare services and help mitigate high medical care costs through improved self-management among African American women living with SLE. The underpinning mechanism of the behavioral change driven by the CDSMP is based on Bandura’s self-efficacy theory, which in turn is derived from the social cognitive theory. People’s perceived efficacy in their ability to produce desired effects by their action may influence their behavior patterns [32]. Moreover, beliefs about individual self-efficacy develop from cognitive appraisal of information that arises from performance, vicarious experience, verbal persuasion, and emotional arousal or physiological feedback [32].

A pilot study conducted by our group found that the CDSMP was accepted by African American women with lupus [33]. Moreover, pre-post intervention quantitative data showed significant improvements in physical health, cognitive symptom management, self-efficacy, communication with physicians, and medication adherence [33]. In this study, we examined whether African American women with SLE perceived that participation in the CDSMP affected their health care engagement behaviors, and the perceived processes through which the program might have enhanced these behaviors.

## Materials and methods

We used a pre-/post-intervention qualitative design to explore our research question with a sample of African American women living with SLE. We chose to use qualitative methods because they excel at capturing perceptions and process [34].

### Participant recruitment

Participants were recruited from the WELL (Women Empowered to Live with Lupus) study, a behavioral trial of the effectiveness of the CDSMP on African American women diagnosed with SLE (Trial registration number NCT02988661). For this study, we conducted a longitudinal quantitative assessment of the CDSMP’s impact on SLE-related outcomes among African American women. WELL randomly sampled 150 African American women from the GOAL (Georgians Organized Against Lupus) cohort; GOAL is representative of the full disease and sociodemographic spectra of those living with SLE in the southeastern USA [35]. We purposively sampled 24 African American women from the WELL cohort to take part in this qualitative substudy. A priori, we set a target sample size of 24 women because we believed we would reach “theoretical saturation” for key themes by the 24th interview (i.e., the point at which no new themes emerge) based on past studies with SLE women about their perceptions of healthcare [36, 37]. Consistent with purposive sampling, the qualitative study sought variation in the sample by factors that might affect responses to the CDSMP, including baseline depressive symptoms, SLE disease activity, age, and education [38–42]. Data on these characteristics were drawn from WELL surveys, which also provided information on sample sociodemographic characteristics.

### Data collection

We conducted two waves of one-on-one, semi-structured interviews with each participant. The first interview (“Wave 1” or “baseline”) was conducted in the month before the participant started the CDSMP; the second interview (“Wave 2”) was conducted 2–6 weeks after the CDSMP had ended. This pre-/post-intervention design allowed us to explore African American women’s perceptions of their self-management behaviors “in real time” before and after participating in the CDSMP, and learn their assessment of the processes through which the CDSMP had affected these behaviors. The semi-structured interview guides are available as Additional file 1.

The Wave 1 guide covered women’s perceptions of (1) the SLE diagnosis experience, (2) SLE symptoms and how they affected women’s health-related quality of life, and (3) health service engagement and doctor/patient communication. This guide was reviewed and piloted with two African American women living with SLE who were on the WELL advisory board; these advisors shared insights into how to strengthen the domains and items. The final Wave 2 guide covered participant perceptions of the (1) workshop and (2) changes in health service use behaviors; when participants did not attend all six sessions, we asked about reasons for missing sessions. When Wave 2 participants only attended one class, interviewers did not probe perceptions of CDSMP impact.

Two trained interviewers (both young adult women), AS and AT, conducted all interviews in a private space accessible to participants. Wave 1 interviews lasted approximately 85 min; Wave 2 interviews lasted approximately 65 min. Interviews were audio-recorded and transcribed verbatim.

### Analysis

Transcripts were analyzed using thematic analysis methods [43], and we searched for variations by participant age, educational attainment, patient-reported depressive symptoms (referred since now as depression), and severity of SLE activity; given that some people did not attend all CDSMP sessions, we also examine variations by the number of sessions attended. Consistent with Lorig’s definition, attendance of four or more sessions was deemed as CDSMP completion [24, 25]. To create a preliminary Wave 1 codebook, the first 3 transcripts were read repeatedly by AS, AT, and HC to develop a list of codes and their definitions. Two analysts (AS and AT) then independently applied these codes to these three transcripts. We then compared coded transcripts. Where inter-coder differences in coding were identified, we discussed the causes of these differences (e.g., unclear definitions, coding errors) and resolved the differences (e.g., clarifying definitions, correcting codes). Thereafter, AT and AS each coded half of all Wave 1 transcripts; to enhance inter-coder agreement, every third transcript was “double coded” by AS and AT, and coding differences were identified and resolved, as described above. The team developed themes through extensive memoing (i.e., writing analytical notes analytical notes capturing thoughts, questions, and/or synthesis of the data).

A Wave 2 codebook was developed and applied through a similar process. Analyses were conducted using MAXQDA version 12.3.2 [44]. As noted, some participants (*N* = 6) only attended one CDSMP session, and interviewers did not probe perceived CDSMP impact. We excluded these individuals from the Wave 2 analysis, unless they volunteered information on perceived CDSMP impact on a particular health service use behavior.

### Ethics

The study was approved by the Emory Institutional Review Board and Grady Health System Research Oversight Committee. All study participants signed informed consent.

## Results

### Participant characteristics

Twenty-four women participated in the baseline qualitative interviews; 23 women also completed Wave 2 interviews. As depicted in Table 1, on average, participants were 48.6 years old (standard deviation [SD] = 13.5) and had been diagnosed with SLE 14 years ago (SD = 8.1). By design, the sample was balanced with regard to education, depression, and SLE severity (disease activity and organ damage). Most participants (91.7%) were currently employed; one third had an annual income of less than $20,000; and about half were currently uninsured or underinsured. Over half (62.5%) of participants were currently in a relationship and about half (54.2%) reported living in the household with a child. Possibly, CDSMP attendance was slightly lower for the 15 women who were currently in a relationship (7/15 attended < 3 sessions vs. 3/9 women who were not in a relationship). Attendance was similar among women who reported living with a child in the household (5/12 attended < 3 sessions) and those who did not (5/13 attended < 3 sessions).

**Table 1:** Demographic information for African American women in the WELL sample (*n* = 24)

Characteristic	Value
Current age, years, mean (range)	48.6 ± 13.5
Age at diagnosis, years, mean (range)	34.5 ± 8.7
Years since diagnosis, mean ± SD	14.1 ± 8.1
Educational attainment, *n* (%)	
Less than high school	2 (8.3)
High school/some college	10 (41.7)
Completed college	12 (50.0)
Depressed, *n* (%)	
No (PROMIS Depression *T*-score < 56)	13 (54.2)
Yes (PROMIS Depression *T*-score > 56)	11 (45.8)
Severity of disease activity score (SLAQ), *n* (%)	
Mild disease activity (score 0–10)	6 (25.0)
Moderate disease activity (score 11–16)	7 (29.2)
Severe disease activity (score ≥ 17)	11 (45.8)
Severity of organ damage score (SA-BILD), *n* (%)	
No organ damage (score 0)	8 (33.3)
Mild organ damage (score 1–2)	8 (33.3)
Moderate to severe organ damage (score ≥ 3)	8 (33.3)
Current household income, *n* (%)	
Less than $20,000	11 (45.8)
$20, 000–$49,000	9 (37.5)
$50,000+	3 (16.7)
Refuse to answer	1 (4.2)
Employment, *n* (%)	
Working full-time or part-time	22 (91.7)
Unemployed/retired/student/homemaker/disabled	2 (8.3)
Insurance status^a^, *n* (%)	
No insurance or underinsured	11 (45.8)
Insured	13 (54.2)
Below 100% poverty^a^, *n* (%)	
No	14 (58.3)
Yes	10 (41.7)
Number of CDSMP sessions attended^b^, *n* (%)	
1–3 sessions	9 (37.5)
4–6 sessions	14 (58.3)
Currently in a relationship, *n* (%)	
Yes	15 (62.5)
No	9 (37.5)
Number of children in household, *n* (%)	
0 children	13 (54.2)
1–2 children	8 (33.5)
3–4 children	2 (8.3)
5–6 children	1 (4.2)

*Abbreviations*: *PROMIS* Patient-Reported Outcomes Measure System, *SLAQ* Systemic Lupus Activity Questionnaire, *SA-BILD* Self-administered Brief Index of Lupus Damage. ^a^Data on insurance and poverty were obtained from the GOAL survey, and all other demographics from the WELL Baseline survey. ^b^One participant completed the pre-intervention interview but did not attend any sessions

### Qualitative findings

The qualitative analysis identified three distinct domains of perceived CDSMP impact: communication with doctors; medication side effect management; and insurance’s effects on healthcare engagement (the latter domain emerged from the transcripts and was not a part of the original guide). We describe findings domain by domain, presenting baseline findings and then post-CDSMP changes within each domain. Six participants only attended one workshop. We did not ask these participants about perceived CDSMP impact because of their slight exposure. Depending on the domain, however, some of these six participants volunteered information about CDSMP impact on health service engagement. When such data were available, we analyzed them and include them in our findings. For the sake of transparency, we note the number of Wave 2 participants who provided Wave 2 data for each domain (see Table 2.

**Table 2:** Summary of patient’s perceived CDSMP impact on health service utilization by domain

Domain	Perceived as satisfactory		Perceived as unsatisfactory	
	Pre-CDSMP	Post-CDSMP*	Pre-CDSMP	Post-CDSMP*
Communication with doctors	14/24	16/18	10/24	2/18
Medication side effect management	3/24	7/17	21/24	10/17
Insured	12/24	14/23	12/24	9/23

*Six participants only attended one session. We did not ask these participants about perceived CDSMP impact because of their minimal exposure. Some of these participants, however, volunteered information about perceived CDSMP impacts on communication, medication side effect management, or insurance

#### Communication with doctors

##### Pre-CDSMP

At baseline, 10 out of 24 participants reported dissatisfaction with their communication with their healthcare providers; 14 women were satisfied with their provider communication (Table 2). Women received care from several providers (e.g., nurses, physician’s assistants, rheumatologists), and in the following sections, women’s accounts may refer to any one, or several, of these healthcare providers. Our analysis indicated that women who expressed dissatisfaction with provider communication tended to be less educated; no differences were observed by age, depression status, or SLE severity.

There were multiple reasons why participants were dissatisfied with their communications with providers: women critiqued providers for having a discourteous “bedside manner;” providing too little information (e.g., about comorbidities, medication); being unapproachable; listening poorly; lacking empathy towards patient’s description of pain; and rushing them. This participant expressed concerns about her provider’s ability to listen to her:

Um, I never really hear them say too often, like, “Do you have any questions or concerns?” I don’t really hear them say that too much. They just kind of just – they just kind of review what you’re on and then they just, you know, start talking about your medicines. Sometimes I wish they’ll listen, like just really listen to you … You know, it’s a short period of time that you’re in and out, I just feel like it’s like, “Okay, you’ve got 10 minutes …”

(Age 46, College graduate, Mild Moderate Lupus, Not Depressed)

Women also worried that their SLE medical history affected their communication with healthcare providers. Some participants were dismayed that providers automatically ascribed any new health problems to SLE. Others were concerned that physicians, regardless of specialty, trivialized non-SLE health problems. One woman disclosed that her rheumatologist had ignored her complaints about symptoms in her finger, attributing them to an ordinary “nail fungus;” because of delayed care, she had to have a partial finger amputation.

Conversely, women (*N* = 14) who were satisfied with their communications with healthcare providers at baseline praised them for sitting with them and taking the time to explain medical jargon, test results, and treatment options, and helping women feel that “no question is too stupid.” These patients reported that they could be transparent about all health concerns and had an agency to discuss their treatment options, refuse new medication, and negotiate changes in dosages. As one participant expressed,

[The rheumatologist] takes the time out to sit down and talk with me for a minute and just try to figure out, like, what’s going on. So he just seems – he seems more sincere to me, because I’ve had it – I’ve been to plenty of doctors where they’re not as great and they don’t care. They spend five minutes in there with you, but that’s why I like … . He listens to me …

(Age 28, College graduate, Mild Moderate Lupus, Depressed)

##### Post-CDSMP

Sixteen out of 18 women reported that the CDSMP helped enhance their communication with doctors; eight of these women had been dissatisfied with communication at baseline and eight had been satisfied (Table 2). Five participants were excluded from the Wave 2 analysis because they only attended one CDSMP session and volunteered no information on communication with providers at Wave 2. Women who reported post-CDSMP improvements in their communication with healthcare providers were more likely to have mild SLE (*N* = 9), or be non-depressive (*N* = 9). Participants who attended 4 or more workshop sessions (*N* = 13) were more likely to report improvements in communication with providers.

Eight out of 10 participants who expressed dissatisfaction with healthcare provider communication at baseline noted improvement in this area after the workshop. Several of these women reported that the CDSMP had taught them to prepare for doctor’s visits using several strategies. Strategies included making lists of their current medications, questions, new symptoms, medication side effects, or other concerns they would like to discuss during the visit. At baseline, only one woman reported making such lists prior to seeing the doctor. List making helped women to remember to ask key questions about their health and care. Discussions at the CDSMP also fostered a desire among women for more open communication between them and healthcare providers regarding their SLE. Women reported that facilitated discussions during the CDSMP about being “rushed” during doctor’s visits empowered them to insist on “*get [ting] what paid for*” by demanding that providers spend sufficient time with them. Two women remained dissatisfied with their communication with healthcare providers (Table 2).

Eight out of 13 women who had reported satisfactory communication with doctors at baseline experienced enhanced communication after the CDSMP. Similar to the improvements reported by participants above, women noted that the CDSMP workshop encouraged them to be more open about their SLE pain and symptoms when communicating with providers and reminded them that they had an agency to ask questions, particularly about new medications. One participant, for example, described how the class had helped her:

Uh, I’ve seen a couple [of doctors], rheumatology, neurology [since completing the CDSMP]. Um, they went fine. I think I was able to speak a little bit more to them. Um, cause I think before [the CDSMP] I was, I didn’t complain as much about the medication not helping. Um, but [now] I have kind of let them know that this [medication], it’s not working for me. None of this stuff [medication] has helped at all. And I’m still in a lot of pain every day. So that’s why they gave me the additional [medication].

(Age 50, College graduate, Severe Lupus, Depressed)

Many women described that they had *known* about these strategies previously, but that the workshop encouraged them to *act* on this knowledge.

Two participants reported no change in doctor-patient communication: one did not recall the session in which this topic was discussed; the other reported that she did not learn anything new about communication from the workshop.

#### Medication side effect management

##### Pre-CDSMP

At baseline, 21 out of 24 women reported experiencing at least one side effect as a result of SLE medications (Table 2). Three participants did not experience any adverse side effects, one because she was not currently taking medications for her SLE symptoms. In the following sections, we only describe experiences of the 21 women who reported at least one medication side effect at baseline. Typically, women suffered from multiple SLE-medication-related side effects at a time; we focus on those side effects that were most commonly mentioned. Our analysis did not reveal patterns regarding women’s experiences by demographic characteristics or health status.

Several women (*N* = 16) reported experiencing weight fluctuations and facial bloating linked to SLE medications. Weight gain was most commonly reported by participants (*N* = 12), although a few participants (*N* = 2) had experienced both weight gain and loss. Four women described facial bloating, commonly referred to as “moon face” or “Prednisone face.” For many, weight fluctuations and facial bloating were the most challenging of all medication side effects reported. Such fluctuations harmed women’s body image, particularly when they gained weight:

I never been a thick person... I never been this size in my life. Before kids I’ve never weighed over 170 pounds full term so I was really skinny … when I started taking the Prednisone, I just blew up like crazy and um I didn’t like that.

(Age 58, High School graduate, Severe Lupus, Depressed)

Many women reported that managing weight fluctuations was outside of their control: “I couldn’t control it [weight gain] at the time.” Other SLE symptoms (e.g., fatigue) and medication side effects often prevented women from engaging in behaviors to manage their weight. One woman mentioned exercising to combat weight gain; however, recent health complications and surgery had prevented her from exercising regularly.

Five women stated that they experienced deteriorating or blurred vision as a result of SLE medications. At least two participants attributed the deterioration of their vision to hydroxychloroquine. Three participants described that they often saw “little spots,” commonly referred to as “floaters,” that interfered with their vision. Women feared that eventually they might lose their vision completely. A participant shared that she avoided driving at night as a result of her poor eyesight and double vision:

… what has changed, and I believe it’s because the medications have changed, is my vision. [long pause] At night, um … I have decreased my driving at night, um. My boyfriend drives more at night. Um, because the best way to explain it, um, I see multiple images of things... So if I’m goin’ down the highway I see trailings of the street signs, or um, cars...

(Age 50, College graduate, Severe Lupus, Depressed)

A few (*N* = 4) participants mentioned that their SLE medications, particularly Prednisone, caused their skin to become hypersensitive and bruise easily. Women had also experienced rashes, typically on the face, arms, and legs. Some reported that these rashes could be “itchy.” Rashes harmed women’s body image and made them feel self-conscious, especially when rashes occurred on their face. To cope, women wore protective clothing, including long-sleeved shirts and long dresses, both to hide rashes from public view and protect their skin from overexposure to sunlight, which could intensify irritations.

##### Post-CDSMP

Seven of the 17 women who experienced side effects at baseline reported that the CDSMP helped them to improve the management of these side effects (Table 2). Four participants were excluded from the Wave 2 analysis because they only attended one CDSMP class and volunteered no information on side effect management at Wave 2. With one exception, women who reported having improved side effect management were older (49.6 years and above) and had attended at least 4 CDSMP classes.

Following the CDSMP, women felt equipped to [1] engage their healthcare providers to either reduce the dosage of medications with overly detrimental side effects, or to explore alternate types of medications; or [2] better accommodate medication side effects. Women discussed three key CDSMP-inspired strategies that aided them to improve medication side effect management. One strategy was to discuss medications with doctors, to review medications they were taking with the doctors regularly and explore alternate medications with less severe side effects, as needed. Another strategy learned from the CDSMP was to adhere to prescription labels and instructions. One woman, for example, learned to identify which of her medications were required to be taken with meals, which in turn had helped reduce her previously persistent stomach irritations. A third strategy women learned from the CDSMP was practicing positivity (i.e., shifting focus away from difficulties with medication side effects to more positive aspects of their lives). While practicing positivity may not have changed the frequency or intensity of women’s medication side effects, it seemed to help them refocus their energies:

I think [with] the lower dosage I don’t see [my facial bloating]. And maybe it’s there. I just don’t see it. Um, I don’t know. It doesn’t bother me. Um, I think if I start to see it would bother me, but I don’t see it. Maybe just fooling myself, but I do have the round Prednisone face, but I just don’t see it, so, again, putting negative thoughts out of my head. If I get a round face I’m healthy, I’m alive, I’m here, I’m not in pain, so, I’m dealing with it … Being around, again, just like a support group. You hear that often enough you really start to believe it and every time I went in there [CDSMP workshop] they’d reinforce what we learned last week or just like you – you’re more than your disease; that you stay up, don’t stay down – don’t - down. Your disease will spiral if you go down. You got to stay up.

(Age 54, College graduate, Mild Moderate Lupus, Not Depressed)

Among the remaining 10 participants (Table 2) who did not report any changes in their management of side effects, 9 reported that they did not learn any useful skills that helped them manage side effects; 1 had stopped taking the medications that caused her side effects.

#### Insurance

Women reported that insurance coverage was a significant factor affecting how they utilized healthcare services and managed their disease; while not probed directly during interviews, this theme emerged organically from women’s discussions of healthcare utilization.

##### Pre-CDSMP

Based on participants’ views, we identified two distinct groups of women at baseline: participants who report ever being uninsured (including those who were currently uninsured; *N* = 12) and participants who were currently insured and did not report ever being uninsured in the past (*N* = 12) (Table 2). Current insurance coverage was strongly patterned by age, education, and disease severity. Women who were older, had fewer years of education, or had mild/moderate SLE were more likely to be uninsured than women who were younger, college educated, or had severe SLE. Many currently insured participants (*N* = 8) were enrolled in either Medicaid or Medicare as part of their social security disability benefits. Notably, five women reported that their SLE diagnosis or symptoms had caused them to lose their jobs and that this job loss in turn caused them to lose their health insurance. Some women worried about their inability to receive approval for disability benefits despite their diagnosis, noting that “… it’s so hard for lupus people to get disability.”

While uninsured, these women used multiple strategies to manage their SLE, including purchasing over-the-counter pain medications that were affordable but often less potent; going into debt to pay for emergency room visits; paying out-of-pocket; enrolling in research studies to obtain blood tests; and seeking care from hospitals that had special policies for the uninsured. Several reported forfeiting needed medications:

… when I first had to leave work … I did not have insurance so I didn’t have um any of my medications

(Age 50, College graduate, Severe Lupus, Depressed*)*

Three participants reported that lack of insurance prevented them from [1] seeking needed medical care from preferred hospitals [2], seeking care from specialists when referred, and [3] obtaining timely care (e.g., some reported wait times up to 3 months). One woman reported,

…with so many appointments that [my primary care physician, and my rheumatologist] want me to go to … I can’t afford to pay all those co-pays. And to go to the rheumatologist or specialty doctors, which is what I mostly have to see lately [coughs] it’s like fifty dollars every time I go to the doctor and I can’t afford that, so it’s kinda tricky.

(Age 45, High School, Severe Lupus, Depressed)

##### Post-CDSMP

The CDSMP did not seem to help women improve their health insurance or better navigate systems for uninsured people. Insurance remained a major barrier to accessing health services:

I wish I learned more about, ah, like say for the people who don’t have the insurance right now, like say I don’t even have the disability and can’t afford to pay for... like one of these medicines is very high, two of them. [I wish] There was more ways that I could find out how to get doctors I need to see for myself … they said you can go to all the free clinics but the free clinics don’t have specialists at their clinic … .they’re not a rheumatologist

(Age 64, High School graduate, Severe Lupus, Not Depressed)

Only three of 23 participants (two belonging to the uninsured group at baseline and one in the insured group) reported any improvement in health insurance coverage or navigation after participating in the CDSMP: two reported that they had started to maximize their use of covered services since Wave 1, and one reported that she had started negotiating with healthcare and insurance providers to receive additional benefits since Wave 1. Two out of these three participants attributed the changes directly to the CDSMP. Our analysis did not reveal patterns in women’s responses by health status, demographic characteristics, or workshop attendance.

## Discussion

The present qualitative study explored whether and how participating in the CDSMP affected healthcare use, from the perspectives of African American women living with SLE. We found evidence that many women in this sample perceived the CDSMP as a valuable resource to improve healthcare use behaviors, including communicating with doctors and managing medication side effects. Many women also volunteered that insurance was one of the key barriers to healthcare use, though the CDSMP did not seem to affect this barrier.

Our analysis suggests that the CDSMP had the most potent and widespread effects on patients’ communication with doctors, according to these participants. Strategies that generated improvements in participants’ ability to communicate with doctors included preparing for appointments (e.g., making a list of questions and medications) and boosting patient participation during doctor’s visits (e.g., increased assertiveness, taking notes, more openness about perceived ineffectiveness of medications or fear of comorbidities). However, improvements were not experienced uniformly across the sample: women with mild/moderate disease severity and non-depressed women were more likely to report improvements in communication with doctors after the CDSMP. These qualitative findings are consistent with prior quantitative data obtained among 698 African American patients with SLE from our GOAL cohort, indicating that suboptimal interactions with providers may be explained by the mental and physical symptoms of the patient [45]. Thus, our data suggest that in addition to standard of care treatment and self-management education, African American patients with more severe SLE activity and depression might need provider-based interventions focused on improving physician-patient communication.

Our analysis also revealed that older women were more likely to report post-CDSMP improvements in medication side effect management. Because older age can potentially increase polypharmacy associated with greater cumulative organ damage [46] and contribute to cognitive impairments to perform instrumental self-management activities in the SLE population [47], our findings support the relevance of the CDSMP program to improve drug side effect management among those at higher risk.

Women who attended 4 or more CDSMP classes were more likely to report improvements in both communication with physicians and management of medication’s side effects, supporting the need of weekly CDSMP workshop encounters with peers for successful self-management skill-building [24, 25].

Although the CDSMP was not designed to improve insurance coverage, our study participants repeatedly noted that insurance was an important barrier to effective utilization of health services. A major cause of loss of insurance is unemployment, which we previously described to occur in nearly 50% of our SLE cohort after 13 years since diagnosis [48]. We also reported that African American patients and those with more severe disease were more likely to be unemployed [48]. Thus, those vulnerable SLE groups are often at a much greater risk of entering in the vicious circle of poverty, lack of healthcare access, and poor outcomes. Our study participants lived in Georgia, a state that has resisted Medicaid expansion, and so under- and lack of insurance remain critical barriers to healthcare services in the state. However, there is a lack of data on the proportion of high-risk individuals with SLE who receive disability benefits and Medicaid coverage in Georgia.

### Strengths and limitations

These findings should be considered in light of the study’s limitations. As with qualitative studies generally, our findings may not be generalizable to the broader population of African American women diagnosed with SLE. However, purposive sampling enabled us to explore variations in themes by participant age, depression, SLE severity, and CDSMP participation. Most GOAL participants have graduated from high school, however, and so we could not achieve our aim of exploring differences in CDSMP experiences by high school graduation status; instead, the sample varies by college graduation, which may be less salient to health care use behaviors and CDSMP experiences than high school graduation status. Further, the study was not able to explore whether or how provider characteristics and responsiveness might shape doctor-patient relationships. Future studies of doctor/patient dyads can explore the effects of physician characteristics on doctor-patient communication. The relationship between the interviewer and the participant can shape multiple dimensions of the research process, including interview guides, participant disclosure, and analyses. In this case, both interviewers were women, neither of whom was living with SLE; one interviewer was Asian American and the other was African American. The team engaged in multiple forms of critical reflexivity about team relationships with participants, including through memos and team discussions. To test the team’s research questions, guides, and interpretations, we partnered with African American women who were living with SLE as we developed the interview guide, and to review and challenge emerging findings.

Our study has several strengths. Each interview was audio-recorded and transcribed verbatim to strengthen descriptive validity (i.e., the data’s accuracy and completeness). To improve interpretive validity (i.e., hewing to participant perspectives), we conducted member checks with two African American women living with SLE, both of whom corroborated our findings. The study’s longitudinal design further enhanced its interpretive validity. We included negative cases to enhance theoretical validity (i.e., considerations of plausible alternate explanations). The study’s longitudinal design, with a high retention rate (95.8%), supported valid comparisons of self-management behaviors reported by participants, pre- and post-intervention.

In conclusion, findings from this novel longitudinal, qualitative study suggest that African American women living with SLE, a population vulnerable to high rates of SLE morbidity and mortality, perceived benefits from CDSMP participation on healthcare engagement. Specifically, our analysis suggests that CDSMP engagement improved their communication with doctors and their medication side effect management. Should additional quantitative studies reach similar conclusions, African American women living with SLE should be encouraged to participate in CDSMP workshops to enhance health service use behaviors, behaviors that have been proven to improve health outcomes among SLE patients. Because the CDSMP is free and widely disseminated across the USA through community centers, it may be a suitable and accessible option for African American women with SLE, even though it is not specifically tailored to SLE.

## Additional file

Additional file 1: WELL Qualitative Study Interview Guide: Wave 1 and Wave 2. (DOCX 41 kb)

## Data Availability

The datasets generated and analyzed during the current study are not publicly available because they are identifiable. Methods to de-identify qualitative data are still under development; stories disclosed during interviews, for example, could easily reveal an individual’s identity when paired with state of residence, SLE status, race/ethnicity, gender, and age. Data are available from the corresponding author on reasonable request.

## Abbreviations

CDSMP: Chronic Disease Self-Management Program
GLR: Georgia Lupus Registry
GOAL: Georgians Organized Against Lupus
SLE: Systemic lupus erythematosus
WELL: Women Living Well with Lupus
