# Supplementary material for: Health service utilization among African American women living with systemic lupus erythematosus: perceived impacts of a self-management intervention
Source: Arthritis Res Ther. 2019 Jun 25;21:155. doi: 10.1186/s13075-019-1942-7 (PMC6593601; doi:10.1186/s13075-019-1942-7)
Supplement: Supplementary file 1 — WELL Qualitative Study Interview Guide: Wave 1 and Wave 2. (DOCX 41 kb) [file 13075_2019_1942_MOESM1_ESM.docx]

**WELL Study Interview Guide – Wave 1**

Thank you so much for talking with me today. This interview should take about 60-90 minutes. This is a ***qualitative*** interview – that may be different from the surveys that you have done before because this will feel more like a conversation.

I am going to ask you questions about living with lupus, and how lupus affects your daily life, how you take care of yourself, and your relationships with your healthcare providers.

If there are any questions that I ask that you don’t want to answer, you can skip the question and we can move onto the next one. It is also OK if you need to end the interview early.

I want to reassure you that this conversation is confidential. We are only using this conversation for research purposes to help women who are living with lupus. What you say will not be part of your medical record at Emory, Grady, any other health facility or any government agency. The only circumstance under which I will have to let someone know about our conversation is if you talk about harming yourself or others just so that we can ensure everyone’s safety. Again, this interview is only for research.

Everything that you say to me today is important. I would like to tape record this interview so that I don’t miss anything that you say. Is that all right with you?

OK let’s get started.

[Turn on the tape recorder.]

**I. Warm up**

1. How long have you lived in Atlanta?

- If moved: what brought you here?

2. What is your favorite thing about living here?

3. What was the last ‘fun’ event you attended?

What made it fun?

**II. Background**

1. How did you first find out that you had lupus?

- When did you learn?

- Who did you learn this from or with?

- How did you first react?

- Who did you tell first? Tell us about that conversation.

- Thinking back over the past x years (years since diagnosis), whose response has been most helpful? Why?
- Least helpful? Why?

2. Can you tell me some of the symptoms you experience due to your lupus?

3. How have your lupus symptoms changed over time?

- Type –
  - Fatigue
  - Pain
  - Skin rash
  - Lupus fog

How would you rate your memory?

How has your ability to remember things changed since being diagnosed?

- Severity – flares
- Which symptoms have been the most challenging for you?
- Do your symptoms vary with the weather?

4. Is there anything else you’d like to share about your symptoms/lupus?

**III. Health-related quality of life**

*Translational text: Now I’d like to ask about how lupus is affecting your quality of life.*

1. How is your lupus affecting your daily activities? For example, driving, errands, grocery shopping, cleaning, cooking, taking care of your kids.

- Probe per symptom
- Describe a typical day when you were flaring?
  - If you attend church, what happens if you are flaring?
- Describe a typical day when you were not flaring?

2. How is your lupus affecting your relationships with your family?

- Probe per symptom

2a. Do you have kids living in the house with you? How is your lupus affecting your relationships

with your kids?

3. Are you currently in a relationship?

4. How is your lupus affecting your relationship with your partner?

0R

4. (If not currently in a relationship: in the past, how did your lupus affect your relationship with your partner(s)?

- Probe per symptom

5. How is you lupus affecting your sexual relationship(s)?

- Probe per symptom

6. How is your lupus affecting your relationships with friends?

- Probe per symptom

7. How is your lupus affecting your work?

- Probe per symptom
- Have you ever missed work because of lupus? Tell me about that.
- Has there been a period of time where you were out of work because of lupus?

Tell me about that.

8. How is your lupus affecting your body image?

- How is lupus affecting your weight?
- Probe per symptom
- How has lupus affected the way your hair looks?
- Probe per symptom
- How has lupus affected the way your skin looks?
- Probe per symptom

6. How does your lupus make you feel emotionally? Why do they make you feel that way?

- Probe per symptom

7. Is there anything else you’d like to share with me about how lupus is affecting your quality of life?

**Mid-point break:** *[Acknowledge that you are halfway through interview and inquire as to whether participant is comfortable so far]*

**IV. Self-Management (Self-Care)**

*Transitional text: Now I want to talk about ways that you take care of yourself – some people call that self-care or self-management.*

1. How do you currently cope with your lupus health problems?

- Pain management
- Sleep/rest (fatigue management)
- Taking medications
- Exercise
- Eating
- Substance use (alcohol, cigarettes, marijuana, misuse of Rx drugs)
- Stress management
- Self-monitoring
- Attending medical appointments
- Attending lupus support groups
- Attending church

2. How has this changed over time?

3. How did you learn that these methods might be helpful?

- Doctor?
- Other health professional?
- Family?
- Friends?

4. How confident are you in using these methods?

5. What makes it hard to take care of yourself?

- What do you do when it gets hard to take care of yourself?
- What helps you take care of yourself?

6. Please describe who makes up your support system.

- In what ways have these people supported you?

7. Who are your best supporters? And why?

- Probe per person (e.g., relationship, role, engagement frequency)

8. Who else have you needed support from but didn’t get?

- In what ways would you have liked them to support you?

9. Is there anything else you’d like to share about how you take care of yourself?

**V. Health Care Services Use**

*Transitional text: Now I want to talk about your experiences with doctors and nurses and health care services.*

1. What kinds of doctors have you seen in the past year to help manage lupus?

- What has stopped you from getting the care you need?
- What has helped you get this care?

2. Thinking about the doctors and nurses who treat you for lupus, who is your favorite? We don’t need a name – you can tell me their specialty instead or make up a name for him or her.

- What do you like about them?
- Can you tell me a story about them that will help me understand why they are your favorite?

3. Thinking about the doctors and nurses who treat you for lupus, who is your *least* favorite? Again, we don’t need a name – you can tell me their specialty instead or make up a name for him or her.

- Can you tell me a story about them that will help me understand why they are your *least* favorite?

1. How do feel about how much your doctors and nurses communicate with each other about your care?

5. Earlier, you mentioned the symptoms you are experiencing now, like… [*list them out as a reminde*r.]

- Which ones have you told your doctor or nurse about?
- Which ones *haven’t* you mentioned to them?

Tell me more about why you didn’t tell them

- What other lupus-related health issues are you *not* getting medical help for?

What is making it difficult for you to get the medical help you need?

6. Have you ever been to the emergency department because of a lupus-related problem?

7a. If so: tell me about your last visit to the emergency department because of a lupus-related problem.

- What happened?
- When did you go?
- What made you decide to go?

7b. If not, how have you avoided having to go to the emergency department because of a lupus-related problem?

8. Sometimes people have to take a lot of medications when they are living with lupus. Thinking about the past month or so have you missed any pills?

- Think about the last time that you missed a pill. Why do you think you missed that dose?
  - - Depression?
    - Fatigue?
    - Stress?
    - Forgot?

9. Sometimes the lupus medicines can have difficult side effects. What side effects have been the hardest for you to live with?

- Have you decided to skip doses because of these side effects?
- Have you ever decided to stop using a medication altogether because of the side effects?
- How did you make this decision?
- Did you make this decision with your doctor or nurse, or on your own?

10. What more could your healthcare provider do to help you?

11. Is there anything else you’d like to share about your experiences with health care providers and health care services?

**VI. Cool down**

1. What else do you think it is important for me to know regarding your experiences with lupus?

2. What do you hope to get out of the Living WELL classes?

- What motivated you to participate in this program?

3. What else would you like to share?

**Thank you so much for sharing your thoughts with me today! I am looking** **forward to talking with you again after you have finished the Living WELL class!**

**WELL Study Interview Guide – Wave 2**

Thank you so much for talking with me today. This interview should take about 60-90 minutes. Again, this is a ***qualitative*** interview – that may be different from the surveys that you have done before because this will feel more like a conversation.

I am going to ask you questions about how the Living Well classes you took about a month ago have affected how you manage your lupus. *I would like to reassure you that this is not a quiz— we only want to understand your experience of the workshop and whether you found it useful.*

If there are any questions that I ask that you don’t want to answer, you can skip the question and we can move onto the next one. It is also OK if you need to end the interview early.

I want to reassure you that this conversation is confidential. We are only using this conversation for research purposes to help women who are living with lupus. What you say will not be part of your medical record at Emory, Grady, any other health facility or any government agency. The only circumstance under which I will have to let someone know about our conversation is if you talk about harming yourself or others just so that we can ensure everyone’s safety. Again, this interview is only for research.

Everything that you say to me today is important. I would like to tape record this interview so that I don’t miss anything that you say. Is that all right with you?

OK let’s get started.

[Turn on the tape recorder.]

**I. Warm Up**

*[Q 1&2 to be used to build rapport if interviewer did not conduct wave 1 interview]*

1. What do you like to do for fun?
2. On a typical weekend, what do you like to do to relax?

*[Start here if interviewer conducted wave 1 interview]*

1. [Anchor: I saw you last in the ___ week of ____(month)]. How has your health been since we last spoke/your first interview?

**II. Background – CDSMP Experiences**

*Transition Text: Let’s start by talking about the classes you attended.*

1. Where was the workshop held?
2. How did you get to the location?

a) What did you think of the workshop location?

1. Overall, what did you think of the workshop/ Living WELL classes?

a) What did you think of the workshop schedule? Day & time

1. What did you learn?
   1. What do you wish you’d learned?
2. Walk me through a typical class.
3. What did you ***like*** about the classes?
   1. Particular parts of the class/particular sessions
   2. Characteristics of other participants (age, gender, chronic disease status of other participants)
   3. Materials provided
   4. [Other] (made friends, snacks, water provided, ease of commute)
4. What did you ***dislike*** about the workshop?
   1. Particular parts of the class/particular sessions
   2. Characteristics of other participants (age, gender, chronic disease status of other participants)
5. Which class was the most useful to you?
6. Which class was the least useful to you?
7. What kinds of people were in your group?
   1. Age
   2. Gender
   3. Chronic disease status
   4. Given these factors, how comfortable were you in the group?

10) Tell me about a time when you shared something personal in the group.

1. How did you feel?
2. How did others react, say?
3. Was there a time that you refrained from sharing something personal? Why is that?
4. Tell me more about the two leaders.
   1. How comfortable were you with the lay leaders?

Their age, gender, manner in which they led the sessions

1. What would you change about the workshop?
   1. Number of people
   2. Age of participants
   3. Age of workshop leader
   4. Gender of participants
   5. Gender of workshop leader
   6. Chronic disease status of participants
   7. Chronic disease status of workshop leader
2. What topics were missing from the workshop?
3. How many classes did you attend?

*Now I’d like to ask about any classes you missed, and the reasons for missing them. You are not in trouble and this will not affect you receiving your incentives for this interview. We want to make sure that future lupus patients can benefit from this class, so your honesty is very appreciated. Your answers will not hurt my feelings.*

1. You missed a few classes. Tell me more about what happened.
   1. Transportation
   2. Not feeling well
   3. Not worth your time
   4. Conflict in schedule
   5. Use what the participant says to probe further (e.g. “It didn’t meet my expectations.” 🡪 “What were your expectations?”)
2. What would have made the class(es) easier/more worth your time?
   1. Probe for more
3. What were your expectations going into the Living Well Workshop?
   1. How were those expectations met?
   2. Not met?

**III. CDSMP & Health-Related Quality of Life**

*Transition Text: Now we’re going to talk about how the classes might have affected your quality of life, including your relationships.*

1. Since we last spoke, have you had a flare?
   1. Tell me about that day/those days.
   2. Did you manage your flares differently than before the workshop?
2. Last time we spoke you mentioned that your **family**… (*key phrases from W1)*

How does your relationship with your **family** now compare to how it was before the workshop?

- 1. In what ways did the workshop affect the relationships you have with your family?
  2. Tell me about a time that this happened. (Probe for a story)

1. Last time we spoke you mentioned that your **kids**… (*key phrases from W1)*

How does your relationship with your **kids** now compare to how it was before the workshop?

- 1. In what ways did the workshop affect the relationships you have with your kids?
  2. Tell me about a time that this happened. (Probe for a story)

1. Last time we spoke you mentioned that your **partner**… (*key phrases from W1)*

How does your relationship with your **partner** now compare to how it was before the workshop?

- 1. In what ways did the workshop affect the relationships you have with your partner?
  2. Tell me about a time that this happened. (Probe for a story)

1. Last time we spoke you mentioned that your **friends** (*key phrases from W1).*

How does your relationship with your **friends** now compare to how it was before the workshop?

- 1. In what ways did the workshop affect the relationships you have with your friends?
  2. Tell me about a time that this happened. (Probe for a story)

1. How do you deal with your emotions **now** compared to before the workshop?
   1. Tell me about a time this happened. (Probe for a story)
   2. Negative emotions?
2. How does the way you feel about your body **now** compare to before the workshop?
   1. Hair?
   2. Skin?
   3. Weight?
3. *If the participant works-* How has the workshop affected your work?
   1. Tell me a story about that. Can you give me an example?
4. What more did you need from the workshop to help your quality of life?
   1. Relationships with family
   2. Relationships with your partner
   3. Relationships with friends
   4. Your emotions
   5. Your body image
   6. Your work

**Mid-point break:** *[Acknowledge that you are halfway through interview and inquire as to whether the participant is comfortable so far. Ask whether the participant wants to take a 5 minute break.]*

**IV. CDSMP & Self-management**

*Transition Text: Now I’d like to talk about how the class might have affected how you take care of the lupus—some call that self-management or self-care.*

1. The last time we talked, you mentioned these are the ways that you cope with your lupus. *[mention key coping strategies from wave 1 interview]*

How do the **strategies you use to cope with your lupus now** compare to how it was before?

- 1. Pain management
  2. Sleep/rest (fatigue management)
  3. Medications for lupus
  4. Exercise
  5. Eating
  6. Stress management
  7. Self-monitoring
  8. Attending medical appointments
  9. Attending other lupus support groups
  10. Attending church

1. Did the class teach you any other ways to cope?

In our first interview we talked about what makes it hard for you to take care of yourself.

(*Mention key points from Wave 1 interview…………..)*

1. How does **the way you take care of yourself now** compare to how you took care of yourself before the workshop?
   1. Can you tell me a story to help me understand how the workshop has changed how you take care of yourself?
2. Tell me about the problem-solving technique that you learned in the workshop that was most useful to you.
   1. Can you describe a day/scenario when you used this?
3. Which members of your support system have you been able to share your workshop/Living WELL classes experiences with?
   1. Tell me about those conversations.
   2. How do they support you now compared to before the workshop? (probe per relationship, role, engagement frequency)
4. What was the most useful communication technique you learned at the workshop/Living WELL classes?
   1. Last time we talked, you mentioned certain people who you needed support from, but don’t get. For example, _________________________ _______________. Do you think you’ll use these tools with those people?
   2. *If participant has used them already:* How did those conversations go? What has been their response? Tell me a story about how that went.

1. In what ways do you still need help with communicating with family, friends and coworkers?
2. What more did you need from this workshop to take care of yourself better?

**V. CDSMP & Health services utilization**

*Transition Text: Now I’d like to ask about how the class might have affected your relationships with doctors, nurses, and the health care system.*

1. Have you seen a doctor since we last spoke?
   1. Tell me about the visit.
   2. Was the visit different because you took the class? Why?
   3. What did you learn in the class that were useful during the visit?
2. Have you been to the ER since we last spoke?
   1. Tell me about what happened.
   2. Was the visit different because you took the class? Why?
   3. What did you learn in the workshop that were useful for the ER visit?
   4. **If no ER visit - did the class help you avoid going to the ER? How?**
3. What did you learn during the workshop that was most helpful for your relationships with your provider?
4. To my understanding, the workshop included a piece about communicating with your doctors and nurses. In what ways did that help?
   1. How will you use those skills in the future?
   2. What other skills do you still need?
5. Last time we talked you mentioned difficulties with medication, such as...

Missing pills,

Did your time in the class affect those issues? How?

1. Last time you talked about side effects like [*insert participant’s answers from Wave 1 interview]*.

How do you think the class changed how you live with those side effects?

**VI. Cool down**

*Transition Text: As we wrap up our interview, I have a few more questions.*

1. What parts of the workshop do you think has had the most influence on your ability to live with lupus?
2. If you were in charge of future workshops, what would you add to make it more helpful for women living with lupus?
3. Is there anything else you’d like to share about the workshop/Living WELL classes?

**Thank you so much for sharing your thoughts with me today!**
